# Fixation of Tripotassium Citrate Flame Retardant Using a Sorbitol and Citric Acid Wood-Modification Treatment

**DOI:** 10.3390/ma17215377

**Published:** 2024-11-04

**Authors:** Sanghun Yun, Adèle Jane Chabert, Holger Militz

**Affiliations:** Department of Wood Biology and Wood Products, Faculty of Forest Sciences and Forest Ecology, University of Göttingen, Büsgenweg 4, 37077 Göttingen, Germany

**Keywords:** wood modification, citric acid, sorbitol, polyesterification, fixation, tripotassium citrate, flame retardant, leaching, bio-based

## Abstract

Wood modification has been explored in various ways to enhance dimensional stability and reduce flammability, with a focus on environmentally friendly treatments to meet market demands. This study aimed to investigate the efficacy of new, potential fire-retardant materials. Specifically, the study examined the combination of tripotassium citrate (TPC), a water-soluble and bio-based fire retardant, with sorbitol and citric acid (SorCA), an eco-friendly thermosetting resin previously studied. While TPC is known to control combustion, its application in wood modification has not been thoroughly researched. To assess the fixation and flammability of these fire retardants, tests were conducted on Scots Pine (*Pinus sylvestris* L.), including chemical analysis, dimensional stability, mechanical properties, flame retardancy, and leaching tests. The combination of SorCA and TPC showed high weight percent gain (WPG) values; however, leaching and anti-swelling efficiency (ASE) tests revealed challenges in fixation stability. The dynamic mechanical properties were reduced, whereas the static strength values were in the same range compared with untreated wood. While TPC exhibited high flame retardancy prior to leaching, its efficacy diminished post-leaching, underscoring challenges in fixation and the need for improved retention strategies. Bunsen burner tests conducted on leached specimens indicated enhanced performance even under severe leaching conditions as per the EN 84:2020 procedure. However, cone calorimetry measurements showed less favorable outcomes, emphasizing the necessity for further investigation into optimizing TPC retention and enhancing treatment efficacy.

## 1. Introduction

Wood, one of humanity’s oldest materials [1], is notable for its natural biomass [2], renewability [3], biodegradability [4], and non-toxicity [5]. It is cost effective, readily available, and possesses a beneficial strength-to-weight ratio [6], making it a central component in architecture and furniture [7,8,9]. However, not all tree species are resistant to natural elements and pests [5,10], presenting challenges that affect their mechanical properties and applications [6,11,12,13,14,15]. Additionally, excessive tropical deforestation has increased wood prices [16] and led to stricter import regulations [17]. This, combined with the increasing emphasis on carbon-neutral wood [5,10], propels advancements in wood-modification techniques [18], such as treatments with preservatives, chemicals, heat, and resin impregnation [16]. However, these methods raise concerns about their toxicity and environmental impact [4,19], driving a market shift toward eco-friendly solutions that reduce hazardous substance emissions [20].

Chemical modification alters wood’s properties through covalent bonding with the cell wall components, either transforming its composition or modifying its properties without changing its structure [21]. The highly reactive hydroxyl group in cell wall polymers causes wood to swell and contract with water content changes [5]. Various modification methods aim to enhance dimensional stability by reducing these hydroxyl groups [22]. Esterification, a common approach, exploits the high reactivity of chemicals with wood cell wall components [23]. Recently, the attention has shifted to bio-based renewable substances like citric acid (CA) and polyols, which are widely used in industries such as textiles, paper, and food [24,25]. Incorporating polyols into CA provides a cost-effective and water-soluble method [6,23,25,26,27,28,29,30,31,32,33,34], facilitating a straightforward and safe impregnation process [4,35]. This approach is gaining traction as a sustainable alternative to traditional chemical wood modifications [4,22]. Among these joint treatments, SorCA (sorbitol and citric acid) has emerged as a noteworthy topic, initiated by researchers like Doll [24] and Larnøy [31]. SorCA utilizes an esterification reaction without a catalyst [4]. Applied to wood via vacuum and impregnation, SorCA undergoes curing to form reactions and esters with cell wall polymers [25], demonstrating positive outcomes in dimensional stability and biological durability [23,31,32,33]. Optimization studies have identified ideal conditions, including solid contents of 30%, a SorCA molar ratio of 1:3, and curing at 140 °C [10,23,24,25,31,36], as applied in this study for resin treatment.

Despite advancements in enhancing wood’s dimensional stability and durability, combustibility remains a significant concern [2,37,38]. Bulk impregnation primarily addresses outdoor applications [39], but the construction industry’s demand for fire-resistant materials underlines the importance of fire safety in public spaces [40,41,42]. High-profile fire incidents, such as the fires at Notre Dame de Paris (2019) and Grenfell Tower (2017), highlight the urgency for effective flame retardancy solutions [43,44,45,46]. Impregnation processes that combine thermosetting resins with compatible fire retardants [11] can mitigate combustion rates and hinder flame spread and protect wood by forming a carbonized layer [47]. While several flame retardants have been studied, concerns about toxicity [41,48,49,50], leaching [51,52], hygroscopicity [53,54,55,56], and unmeasured risks and expenses persist [57,58]. The challenge remains to develop bio-based, sustainable, water-based, and affordable flame retardants that are effective in fixation and meet safety standards [55,59]. A recent study by Kurkowiak [10] has explored the combination of phosphorus-based flame retardants with SorCA, demonstrating improved fixation of highly leachable flame retardants. Similarly, prior research by Wu [11,37] also investigated phosphorus-based flame retardants paired with non-bio-based curing resins, yielding positive results in flame retardant fixation, leading to the proposal of studying flame retardant fixation with the curing resin SorCA.

This study introduces tripotassium citrate (TPC) as a novel additive for enhancing flame retardancy in wood. Tripotassium citrate (TPC) or potassium citrate (PC) is commonly used in medical treatments for kidney stones [60,61,62] and in various tobacco and fire industries. Among the additives for combustion regulation, potassium citrate effectively controls pyrolysis [63,64,65,66,67,68]. Additionally, potassium citrate is used in K-grade fire extinguishers for greasy fires [69]. This expandable effect is utilized across various fields and is environmentally friendly [48,70,71,72].

SorCA has been well researched for its durability benefits against fungi, offering a fine, non-toxic, and bio-based wood-modification method, although it lacks flame-retardant properties. Therefore, the combination of TPC with SorCA represents a unique approach to wood modification, potentially providing both durability and flame retardancy in a sustainable manner. Most previous research has focused on phosphorus-based or non-bio-based flame retardants, which, despite their effectiveness, raise concerns about their environmental impact, toxicity, and limited long-term stability.

Thus, this study aims to evaluate the effectiveness of the novel joint treatment of tripotassium citrate (TPC) and SorCA (sorbitol and citric acid) for wood modification, highlighting its unique contribution to the field of wood modification, which has not been previously addressed in the existing literature.

## 2. Materials and Methods

### 2.1. Chemical Reagents and Wood Materials

Technical-grade sorbitol (approx. 98% purity) was produced by Ecogreen Oleochemicals GmbH (Dessau-Roßlau, Germany), food-grade citric acid (approx. 99.5% purity) was produced by Laiwu Taihe Biochemistry Co., Ltd. (Jinan, China), and food-grade tripotassium citrate (approx. 99% purity) was provided from Jungbunzlauer Ladenburg GmbH (Ladenburg, Germany), and they were employed in this study. TPC of 10%, 20%, and 30% input, relative to the total proportion of the SorCA solution, was added. Table 1 illustrates the solution formulation and pH value. All solutions were prepared under laboratory conditions. The mixing sequence involved around 30 °C tap water, and the pH values were measured using a WTW inoLab Multi 9210 IDS (Xylem Analytics Germany Sales GmbH & Co., KG, Weilheim, Germany).

Scots Pine (*Pinus sylvestris* L.) sapwood from Lower Saxony, Germany, was cut in radial (R), tangential (T), and longitudinal (L) directions for each experiment. The dimensions and repetition of the specimens are shown in Table 2.

### 2.2. Modification Process

The specimens were dried in an oven prior to impregnation. Specifically, specimens sized 20 × 20 × 10 mm^3^, 10 × 10 × 160 mm^3^, and 4 × 13 × 125 mm^3^ were dried at 103 °C for 24 h, and specimens sized 20 × 90 × 250 mm^3^ and 18 × 100 × 100 mm^3^ were dried for 72 h. A vacuum was then applied for 30 min with a pressure range from −960 to −980 mbar. Following the vacuum step, impregnation was carried out for 2 h under pressures ranging from 10,000 to 10,800 mbar. After impregnation, the excess formulation on the surface of the specimens was wiped off with tissue paper before measuring the solution uptake (SU) and air drying at room temperature. All specimens were then dried at room temperature for 24 h before being cured in an oven at 140 °C for 24 h.

### 2.3. Dimensional Stability

For dimensional stability measurements, specimens sized 20 × 20 × 10 mm^3^ were used, with 10 specimens from each collective. The mass and dimensions of the specimens were recorded in four states: oven-dry condition, immediately after impregnation, post-curing, and after saturation in water. For the measurement of mass, electronic devices such as a CP323 balance (Sartorius AG, Göttingen, Germany) with an accuracy of ±0.001 g were used, and for dimensions, a digital indicator S dial 100 caliper (Sylvac, Yverdon, Switzerland) with an accuracy of ±0.01 mm was used.

#### 2.3.1. Solution Uptake (SU)

Solution uptake (SU) was calculated according to Equation (1). It represents the specimen’s mass change between being oven-dried and immediately after impregnation.

(1) $$SU\;\%=\frac{M2-M1}{M1}\cdot 100$$

Here, M1 = the mass after oven drying (anhydrous state), and M2 = the mass after impregnation.

#### 2.3.2. Cell Wall Bulking Coefficient (CWB)

The cell wall bulking coefficient (CWB) was calculated according to Equation (2). This represents the changes after impregnation and curing, reflecting the modifications from the non-leaching treatment state. The value is derived from radial and tangential (RT) measurements, indicating changes in the internal structure of the wood.

(2) $$CWB\;\%=\frac{W2-W1}{W1}\cdot 100$$

Here, W1 = RT after oven drying (anhydrous state), and W2 = RT after curing (anhydrous state).

#### 2.3.3. Weight Percent Gain (WPG)

The weight percent gain (WPG) was calculated using Equation (3). This represents the mass change between the oven-dried state and the specimen after curing, indicating the amount of chemical retained in the specimen. WPG is an important indicator of the chemical treatment’s effectiveness. It can also be measured after a leaching treatment, depending on the intended use of the wood.

(3) $$WPG\;\%=\frac{M3-M1}{M1}\cdot 100$$

Here, M3 = the mass after curing (anhydrous state).

#### 2.3.4. Leaching Rate (LR)

Leaching analysis is vital for assessing the TPC and SorCA treatments’ long-term effectiveness in outdoor conditions. High leaching rates can diminish flame retardancy and durability, impacting their industrial viability and safety.

In this study, the leaching rate was calculated according to Equation (4), following the EN 84:2020 procedure [73]. This process shows the change in mass between curing and post-leaching. After leaching, the specimens were oven-dried and compared with their mass after curing. The cured specimens were then immersed in distilled water and placed in the autoclave. A vacuum was applied for 30 min, with pressures ranging from −960 to −980 mbar. Following the vacuum process, the distilled water was changed 10 times over 14 days. Post-leaching, the specimens were sequentially dried at room temperature for 72 h, then in a 60 °C oven for 24 h, in an 80 °C oven for 24 h, and in a 103 °C oven for 24 h. After drying, the specimens were measured, and fire-test specimens were stored in a conditioning room at 20 °C and relative humidity (RH) of 65%.

(4) $$LR\;\%=\frac{M3-M4}{M3-M1}\cdot 100$$

Here, M4 = the mass after leaching (anhydrous state).

#### 2.3.5. Anti-Swelling Efficiency (ASE)

The anti-swelling efficiency (ASE) was measured according to DIN 52184:1979 [74]. The oven-dried specimens after EN 84:2020 leaching were immersed in distilled water and placed in an autoclave. A vacuum was applied for 30 min, with pressures ranging from −960 to −980 mbar. After the vacuum process, the specimens were kept in distilled water for 24 h. To measure the wet specimens, the surface moisture was wiped off with tissue paper before recording their weight and dimensions. The specimens were then sequentially dried at room temperature for 72 h, followed by 24 h in an oven at 60 °C, 80 °C, and 103 °C, respectively. This process was repeated five times to obtain various coefficients. The anti-swelling efficiency (ASE) was calculated using Equations (5)–(7).

(5) $$Sr\;\%=\frac{Vr,sat-Vr,d}{Vr,d}\cdot 100$$

Here, Sr = the total swelling measured on the untreated specimen; Vr,sat = the volume of the untreated specimen after immersion into water; and Vr,d = the reference volume in the anhydrous state.

(6) $$St\;\%=\frac{Vt,sat-Vt,d}{Vt,d}\cdot 100$$

Here, St = the total swelling measured on the treated specimen; Vt,sat = the volume of the treated specimen after immersion into water; and Vt,d = the volume of the treated specimen in the anhydrous state.

(7) $$ASE\;\%=\frac{Sr-St}{Sr}\cdot 100$$

#### 2.3.6. Equilibrium Moisture Content (EMC)

To evaluate the mechanical properties and flame retardancy, specimens after curing and specimens after leaching were placed in an environment with a temperature of 20 °C and a relative humidity of 65% for at least 3 weeks. Mass changes were then measured. Equations (8) and (9) were used to calculate the equilibrium moisture content after curing and after leaching, respectively.

(8) $$EMC\;ac\;\%=\frac{M5-M3}{M3}\cdot 100$$

(9) $$EMC\;al\;\%=\frac{M5-M4}{M4}\cdot 100$$

Here, EMC ac = the EMC after curing; EMC al = the EMC after leaching; and M5 = the mass after conditioning (at equilibrium).

### 2.4. Flame Retardant Measurement

All specimens underwent leaching according to EN 84:2020 [73] regulations. They were then conditioned for at least 3 weeks in a climate room set to a temperature of 20 °C and a relative humidity of 65%. The moisture content within the specimens was determined by comparing their mass with that of dried specimens after leaching. Specimens that were not leached according to EN 84:2020 were also conditioned for at least 3 weeks, and the moisture content was measured by comparing their mass to that of the cured specimens.

#### 2.4.1. Burner Test

The burner test, introduced by Pries & Mai [75], provides an expedited assessment of fire retardancy. A Bunsen burner DIN (NG-2411 BO0031) from Juchheim Laborgeräte GmbH (Bernkastel-Kues, Germany) and propane gas were used for this test. Specimens measuring 4 × 13 × 125 mm^3^ were mounted on a fixture at a 45-degree angle, with the lowest of the specimen positioned at the center of the burner. The vertical distance between the burner and the specimen was 8 cm. During the test, the time from ignition to flame-off was measured, with the ignition lasting for 30 s. The gas was turned off after 30 s to cease ignition. The specimens were weighed continuously throughout the test, including during ignition and after flame-off. The flame-off time, weight loss, and weight change at each 10 s interval were recorded. Equation (10) was used to calculate the mass loss before and after the test. Five specimens were tested for each type of collective to calculate the mean value.

(10) $$ML\;\%=\frac{Mab-Mbb}{Mbb}\cdot 100$$

Here, ML = the mass loss; Mab = the mass after burning; and Mbb = the mass before burning.

#### 2.4.2. Single-Flame Test

The single-flame test was conducted using Netzch’s KBK 917 (Taurus Instruments AG, Weimar, Germany) and propane gas (propane gas: ≥95% purity; gas pressure: 4.0 bar), following the ISO 11925-2:2020 [76] procedure. A specimen sized 20 × 90 × 250 mm^3^ was prepared, with a line drawn at 40 mm along the longitudinal direction on the tangential plane. An additional line was drawn 150 mm above. The igniter was positioned so that the flame could ignite at the 40 mm line, with the flame height set to 20 mm and the igniter adjusted to a 45-degree angle. The test was conducted with an ignition time of 120 s. While ISO 11925-2:2020 originally stipulated a 30 s ignition time, the test duration was adjusted due to the larger volume and mass of the wood specimens compared to the material suggested by the standard. The 30 s test time was insufficient to discern differences between treated and untreated specimens. During the test, the ignition of the specimen itself, fire extinguishing time, and combustion range exceeding 150 mm were measured. Two specimens were tested for each type to calculate the mean value.

#### 2.4.3. Cone Calorimeter

The cone calorimeter used was an FTT0014 MLC 11325 (MLC FTT, Fire Testing Technology, East Grinstead, UK) and followed the procedure outlined in ISO 13927:2015 [77]. Specimens sized 18 × 100 × 100 mm^3^ were exposed for 30 min at an external heat flux of 50 kW/m^2^. The distance from the cone to the specimen was set to 25 mm, and the temperature of the cone heater was maintained at 778 °C. The igniter, positioned at the center, ignited the specimen, which was covered with aluminum foil to expose only the top surface to the fire. During the test, parameters such as the heat release rate (HRR), peak heat release (PHR), time to peak heat, total heat release (THR), mass loss, ignition time, and flame-off time were calculated automatically using the MLCCalcprogram. Five specimens were tested for each type to compute the mean value.

### 2.5. Mechanical Properties

A ZO10 10 kN (Zwick GmbH & Co. KG, Ulm, Germany) testing machine was employed for three-point bending tests. Additionally, a Resil Impactor 25J (CEAST GmbH, Martinsried, Germany) was used for impact bending strength tests. All specimens underwent a curing process in an oven at 140 °C for 24 h after impregnation. Subsequently, they were conditioned for a minimum of 3 weeks in a climate-controlled room set to a temperature of 20 °C and a relative humidity of 65%, without leaching. The moisture content in the specimens was then determined through mass comparisons with the cured specimens. The mechanical properties, including the modulus of rupture (MOR), the modulus of elasticity (MOE), and the impact strength properties, were evaluated.

#### 2.5.1. Three-Point Bending Test

The three-point bending test was conducted in accordance with the DIN 52186:1978 [78] standard. Specimens measuring 10 × 10 × 160 mm^3^ were employed for the test. A test speed of 1.7 mm/min was utilized, and the test continued until failure occurred. The unloading point of the machine was reached when the maximum force decreased by 20% following loading to the specimen’s break point. Twenty specimens were tested per collective and control. The maximum force at which failure occurred (Fmax) was recorded during the test. The modulus of elasticity (MOE) and modulus of rupture (MOR) were then calculated using Equations (11) and (12). The test values were calculated automatically using the TestXpert program. The mean value for each group was calculated using 20 specimens.

(11) $$MOE=\frac{l^{3}}{4\cdot b\cdot h^{3}}\cdot \frac{\Delta F}{\Delta f}$$

(12) $$MOR=\frac{3\cdot Fmax\cdot l}{2\cdot b\cdot h^{2}}$$

Here, l = the span length between supports (mm); b = the width of the specimen (mm); h = the depth of the specimen (mm); ΔF = the change in applied force; Δf = the change in deflection at the midpoint of the specimen; and Fmax = the maximum force applied (N).

#### 2.5.2. Impact Bending Strength Test

The impact bending strength test was conducted following the procedure outlined in DIN 52189:1981 [79]. The span width was 105 mm. Twenty specimens per group, each measuring 10 × 10 × 160 mm^3^, were tested. The mean value was calculated based on these specimens. Equation (13) was employed to calculate the impact value:

(13) $$w=\frac{1000\cdot W}{a\cdot b}$$

where w = the impact strength in kJ/m^2^; W = the work required to break the specimen (total energy) in J; and a·b = the specimen dimensions (tan and rad) in mm.

### 2.6. Chemical Property Analysis

Fourier-transform infrared (FTIR) spectrum analysis of the polymers used in the wood was conducted using an Invenio S spectrometer (Bruker optics GmbH & Co. KG, Ettlingen, Germany) with an attenuated total reflection (ATR) unit in a range of 4000~400 cm^−1^, at a resolution of 4 cm^−1^, and with 64 scans using ATR mode. Baseline correction and normalization were processed using OPUS 7.5 software. The treated specimens were scraped and measured. These specimens were not subjected to EN 84:2020 leaching before measurement.

## 3. Results and Discussion

### 3.1. Dimensional Stability

#### 3.1.1. Solution Uptake (SU)

**Figure 1:**
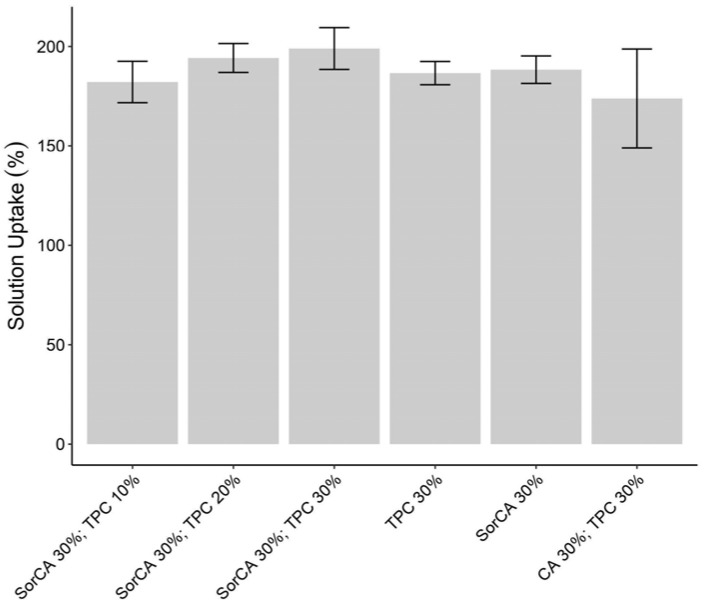
Solution uptake (%) after impregnation.

Solution uptake (SU) serves as the initial indicator of suitability for impregnation. Solution absorption was calculated based on mass. Figure 1 shows that all solution absorption rates exceeded 100%. The lowest absorption rate was approximately 170%, which is considered sufficient and suitable for absorption [31]. This level is comparable to the absorption rate with water when all the cell wall pores and free lumina of the wood are saturated [80]. Therefore, sufficient impregnation with all the chemicals was achieved.

However, SU alone does not indicate whether the wood was cross-linked with impregnation solutions. It is also possible that it absorbed water. Therefore, it is necessary to determine the fixation of the chemical(s) after curing, which is the second step of the thermosetting resin process. Effective fixation is achieved by optimizing process parameters such as the amount and ratio of the chemicals and the curing temperature [23,24,25,26,31,36,81].

#### 3.1.2. Cell Wall Bulking Coefficient (CWB)

Cell wall bulking was calculated using the transverse (radial and tangential) section. Thus, it indicates the extent to which the cell wall swells, signifying that the cell wall remains swollen after curing [82,83]. This change is due to the bulking of the precipitated chemicals [2,6], which is expressed as an increased volume in the specimen under oven-dry conditions. Dimensional stability can be improved by bulking once the molecules of chemicals are larger than water molecules, which are deposited in the cell wall. This permanently expanded cell wall stabilizes the dimensions by reducing the water-soluble space of the cell wall matrix [84].

**Figure 2:**
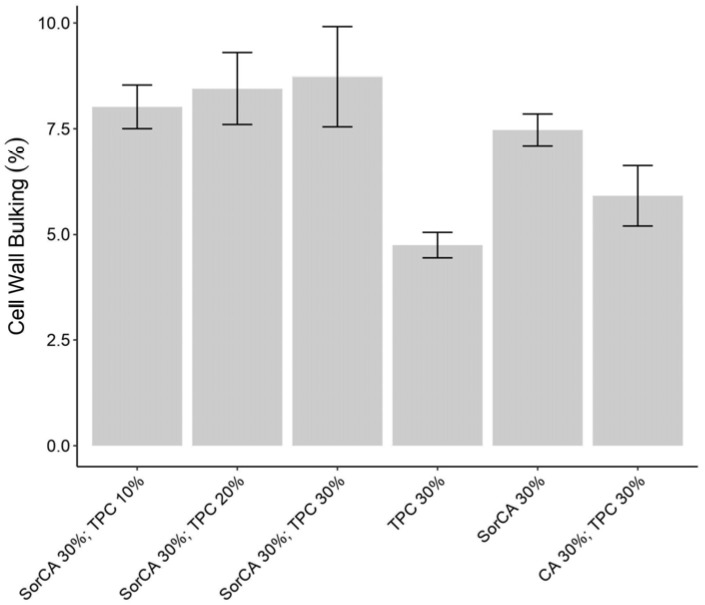
Cell wall bulking (%) after curing at 140 °C for 24 h.

The cell wall bulking (CWB) results reveal that solutions with SorCA show higher values than the non-SorCA specimens, as seen in Figure 2. The values for SorCA were similar to those reported in other studies [10,25], but further investigation is needed for the high bulking seen, where TPC is added to SorCA. Although there was no significant difference, it appears that, like in the SorCA study, the bulking value also increased with the amount of TPC added [25].

Stamm and Seborg [85] identified criteria for effective resin treatment, including a molecular size small enough to penetrate cell walls, the solubility of molecules, and affinity with cell walls. Imamura [86] further revealed that the average molecular weight of the impregnation agent affects the depth of penetration into the wood. In future studies, a molecular unit intracellular penetration study for combinations of SorCA and TPC is therefore proposed.

#### 3.1.3. Weight Percent Gain (WPG)

The weight percent gain (WPG), a value obtained by comparing the mass after impregnation and curing to the oven-dry mass, indicates how much of the treatment remains in the wood [25]. It reflects the efficiency of the treatment process in terms of the retention of the added chemical and can vary due to different parameters such as chemical properties and wood species [82].

**Figure 3:**
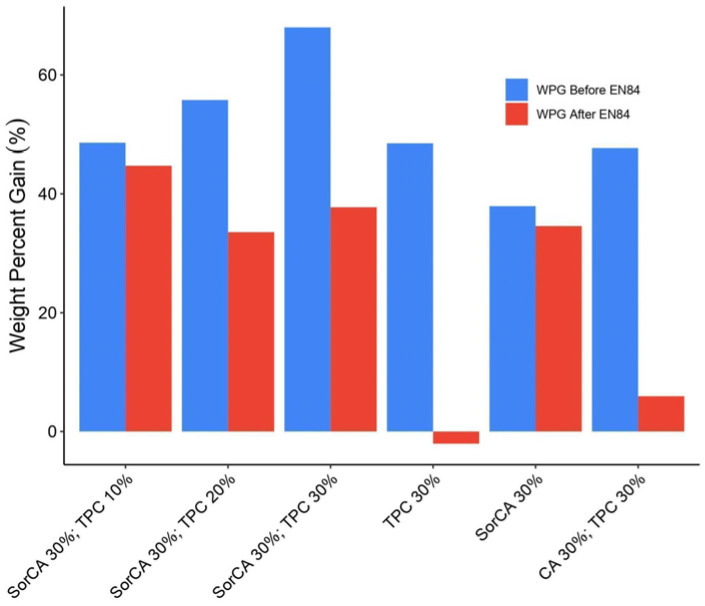
Weight percent gain (%) before and after EN 84:2020 leaching procedure.

By examining the WPG before leaching in Figure 3, we observe that the treatments reached WPG values between 35 and 70% depending on the type of chemical and the concentration. While this generally aligns with the CWB graph (Figure 2), the results for TPC 30% combined with SorCA 30% and CA 30% combined with TPC 30% differ when considering the correlation between CWB and WPG [24,25,36,87]. This discrepancy may be due to the deterioration of wood components under acidic conditions, although such phenomena typically occur at high temperatures [25]. Alternatively, the addition of TPC might require higher temperatures and longer durations for complete polycondensation, resulting in retained water in the wood and, thus, higher WPGs [23,26,31,81].

The after-leaching values represent the WPG values post the EN 84:2020 procedure, which is essential for evaluating the fixation of treatment agents in externally exposed wood [82]. A smaller difference in WPG values before and after leaching indicates better leaching resistance of the added chemicals [37]. Solutions including SorCA showed relatively smaller differences compared to those without SorCA. The negative value after leaching in TPC 30% probably indicates that leaching not only of the largest amounts of TPC itself but also of the wood extractives and other degraded wood components occurred. The mean leaching value for the untreated specimens was 2.8% with a standard deviation of ±0.3%. The negative value for TPC 30% is considered equivalent to the extract value, aligning with the typical leaching range of 3 to 5% for water-soluble extracts from wood. For a clearer view of the actual leaching substances, the leachate can be chemically analyzed in future studies, as studied by Lin [82].

As a result, flame retardants not bound to SorCA were not fixed well in the wood. Although the fixation of CA itself has been extensively studied [24,88,89,90,91], the low immobilization of TPC without sorbitol suggests a need for additional research. Moreover, a CA-only group (without sorbitol) was not included in this study design, leaving further comparisons for future investigation.

#### 3.1.4. Leaching Rate (LR)

In chemical wood treatment, the number of impregnated products plays a crucial role in imparting new properties to the wood [55]. For exterior materials exposed to water, adequate internal fixation of the impregnation solution is essential; otherwise, the benefits of chemical treatment are compromised [3,55]. Therefore, the EN 84:2020 [73] leaching procedure evaluates the extent of cell wall fixation of treatments assuming exposure to external environmental conditions.

**Figure 4:**
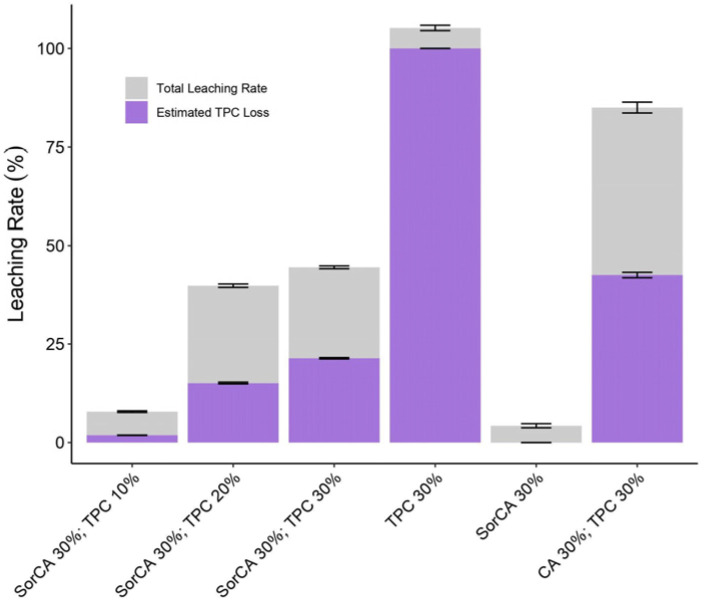
Leaching rate (%) and estimated loss (%) of TPC.

Figure 4 illustrates the mass change of the cured specimens following the EN 84:2020 leaching procedure. The value, much like the WPG, varies according to the presence of SorCA. For TPC 30%, it appears that both the added solution and the wood extractives were leached [6]. This observation suggests that the ionic nature of TPC, which contributes to its high solubility in water, could be a significant factor leading to its poor retention. Without forming strong covalent bonds with the wood cell walls, TPC remains vulnerable to leaching in moist conditions.

During leaching, some single-flame and cone calorimeter specimens developed surface fissures and deeper cracks. These defects could be attributed to shifts in the hygroscopicity, irregular swelling, variations in reagent concentration, pH fluctuations, and alterations in the wood’s structure [92]. Such structural changes may further destabilize the wood, enhancing increased leaching of TPC.

Although the inadequate fixation of flame retardants has been widely documented, some studies have observed that flame-retardant effects can still persist even after significant leaching [3,6,10,11,37,41,55,82]. However, with prolonged external exposure, complete leaching of the treatment agent remains a concern, highlighting the need for further research into optimizing surface coating treatments or enhancing the polymerization reactions to improve retention. Developing more robust fixation techniques, such as integrating cross-linking agents or surface modifiers, could be crucial in improving the performance and durability of TPC in practical applications.

The TPC values shown in the graph are theoretical estimates that are calculated by inversely arithmetically determining the ratio of TPC injected during impregnation to the leachate for each group. For example, TPC 30% impregnated solely with TPC shows 100% TPC leached, with additional leaching potentially from wood extractives, resulting in an estimated leaching rate of 105%. Unlike SorCA 30%, which did not contain TPC, no TPC leachate was shown. Several factors contribute to leaching, including high chemical concentrations [25] or inadequate polymerization and covalent bonding due to inappropriate curing temperatures [31,55]. Moist conditions further complicate fixation [93], and hygroscopic salts may be removed by water movement during leaching [3]. To accurately determine the exact leaching amount of TPC, direct analytical methods, such as inductively coupled plasma (ICP) spectroscopy, could be employed [82]. These methods allow for precise quantification of the leached potassium ions from TPC, providing a more accurate assessment of its retention and leaching behaviors.

#### 3.1.5. Anti-Swelling Efficiency (ASE)

The anti-swelling efficiency (ASE) measures the resistance of wood to swelling caused by moisture. The higher ASE values indicate greater dimensional stability, meaning less changes in wood dimensions due to moisture [94]. The ASE was measured after the EN 84:2020 leaching procedure, assessing the volume changes when the wood was fully wetted and subsequently dried over five cycles of measurements.

**Figure 5:**
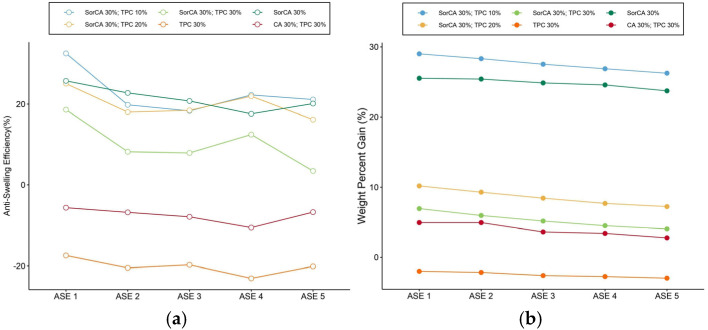
(**a**) ASE (%) variation in 5 cycles; (**b**) WPG (%) variation in each ASE cycle.

Figure 5a displays the initial ASE results: specimens with SorCA recorded values between 20% and 30%, while those without SorCA demonstrated negative values. These negative values indicate very low dimensional stability compared to typical ASE values, suggesting unstable cell wall fixation of the treatment agent. During the second ASE process, leaching was still observed in the SorCA and TPC combination post-EN 84:2020, and the recorded value was marginally higher than the other specimens.

Similar to the WPG values before and after leaching, smaller fluctuations in the ASE values across cycles indicate higher dimensional stability. SorCA 30%, treated solely with SorCA, exhibited the greatest stability, while TPC 30% combined with CA 30% and solely TPC 30% were unstable, showing negative ASE values. However, SorCA 30% remains relatively low when compared to other SorCA studies [4,31,36]. Specimens treated with SorCA and TPC demonstrated low dimensional stability due to significant fluctuations across the ASE cycles, indicating unstable cell wall fixation of the treatment agent. SorCA 30% combined with TPC 10% showed tendencies similar to SorCA 30% from the 2nd to 5th cycles, suggesting potential fixation of only SorCA, although this conclusion requires further verification.

Figure 5b correlates the WPG measurements with each ASE cycle. SorCA 30% demonstrated stability in both the leaching rate and ASE, and SorCA 30% combined with TPC 10%, showed a similar trend and exhibited relatively high values, indicating the fixation of the treatment agent within the wood, contributing to dimensional stability. Contrarily, poorer fixation of the treatment agent correlates with lower dimensional stability. While the exact leaching amount of TPC remains uncertain, SorCA 30% with TPC 10% appears relatively well fixed, as inferred from the higher WPG and ASE values compared to SorCA 30%. However, the possibility that all TPC washed away cannot be dismissed, leaving SorCA 30% with TPC 10% as SorCA-treated wood with values higher than SorCA 30%.

In conclusion, CWB, WPG, and ASE are interconnected. Greater chemical penetration into the cell wall and polymer absorption increases the cell wall volume, thereby enhancing dimensional stability by reducing water absorption [95]. However, a higher WPG does not uniformly indicate better wood properties. The potential for intracellular cracking has also contributed to a decrease in the ASE at high WPG levels [12,96,97,98].

The ASE indicates that dimensional stability varies. For instance, while SorCA treatment can enhance dimensional stability, it may also lead to excessive expansion due to additional sorption sites from untreated chemicals [25,99].

#### 3.1.6. Equilibrium Moisture Content (EMC)

**Figure 6:**
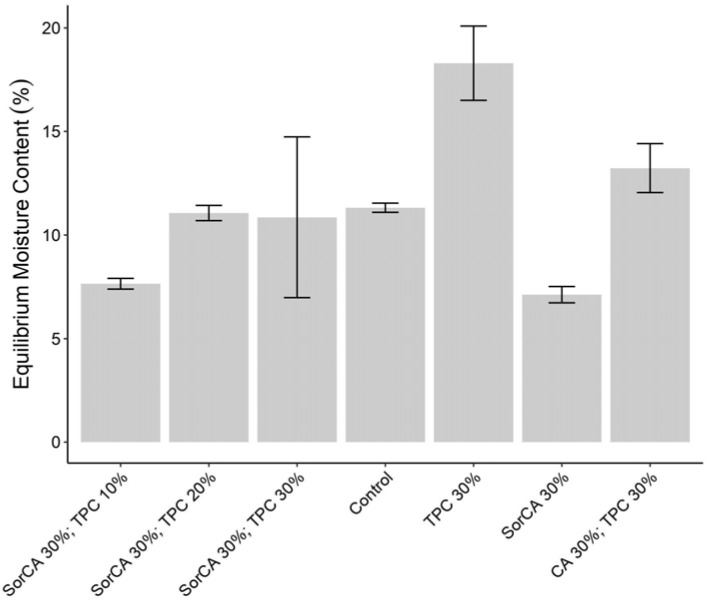
EMC (%) after conditioning at 25 °C and 65% RH for a minimum of 3 weeks.

Figure 6 depicts the equilibrium moisture content (EMC) measurements obtained using impact bending strength test specimens. It was measured after conditioning at 25 °C and 65% relative humidity for a minimum of 3 weeks. The control, untreated wood, exhibited a typical EMC value of approximately 12% [16]. Specimens of SorCA 30% with TPC 10% and SorCA 30% showed relatively low EMC values, indicating improved dimensional stability. This improvement is attributed to the effective cell wall fixation of the treatment agent, which reduces the moisture content by limiting access to hydroxyl sites [6,30]. Studies by Altgen [100] and Kurkowiak [25] support this observation, noting a decrease in water-accessible space. These results align with the WPG and ASE findings [25].

Conversely, EMC values similar to, or higher than, those of control specimens suggest reduced fixation of the treatment agent, indicating it may not be functioning effectively. TPC 30% and CA 30% with TPC 30% exhibited relatively high EMC values, possibly due to an increase in -OH groups from incomplete treatment reactions [25].

However, the high EMC values of TPC 30% and CA 30% with TPC 30% do not necessarily indicate a negative impact on their mechanical properties. For untreated wood, the EMC directly influences the mechanical properties and flame retardancy because moisture causes swelling in the cellulose fabric of the cell wall, weakening the bonding [18,54,101]. In contrast, treated wood is influenced by the deposition of chemicals. When moisture accumulates in the lumen, the cell wall remains structurally unaffected, thereby maintaining its function [16].

**Figure 7:**
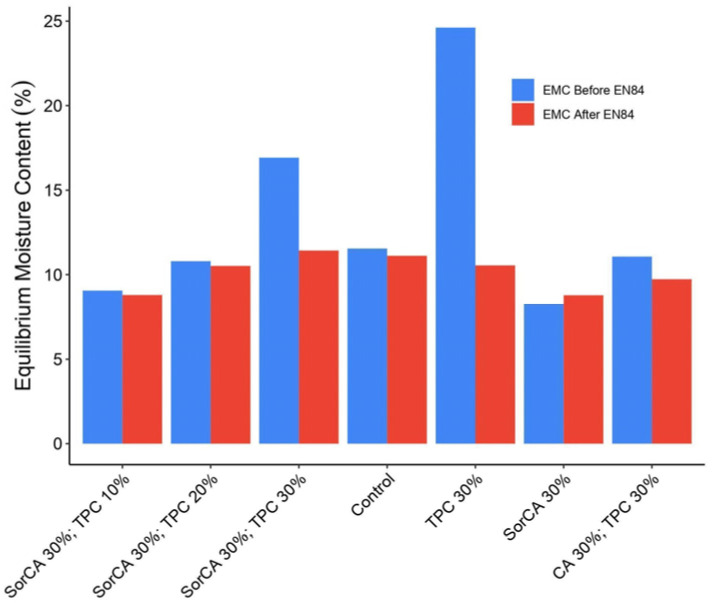
EMC (%) changes in burner test specimens before and after EN 84:2020.

There was a marked difference in moisture absorption between non-leached and leached specimens in Figure 7. TPC 30% experienced substantial chemical loss after leaching. SorCA 30% with TPC 30% also showed decreased moisture content post-leaching, while the other specimens maintained consistent values.

### 3.2. Flame Retardant Measurement

#### 3.2.1. Burner Test

**Figure 8:**
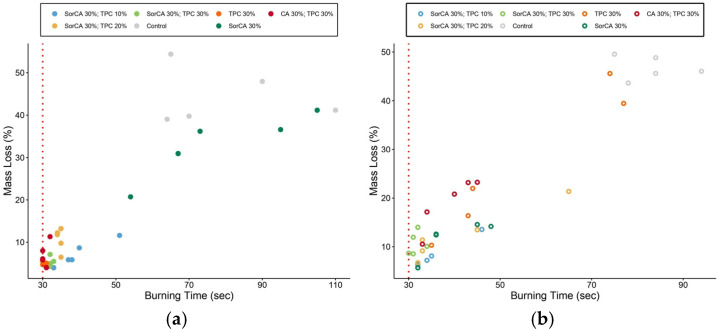
Burner test results (**a**) before and (**b**) after EN 84:2020 procedure. The test has a combustion time of 30 s. Therefore, the specimen’s self-combustion begins after 30 s. If the specimen self-extinguishes at 30 s, it does not ignite.

In Figure 8a, TPC 30% showed the highest immediate resistance. Specimens with TPC demonstrated a self-extinguishing behavior by forming a char layer upon exposure, contrasting with untreated wood [37].

Similarly, Figure 8b displays post-leaching specimens and shows untreated wood to be the least fire resistant. Significant fluctuations were observed in TPC 30%, where leaching was most severe. CA 30% with TPC 30% exhibited a similar pattern, while SorCA 30% showed improved flame retardancy after leaching, potentially due to increased water absorption marginally. Further investigation is needed to understand the unexpected increase. Although SorCA- and TPC-integrated specimens experienced a decline in their fire-retardant properties after leaching, they did not exhibit the significant fluctuations seen in TPC 30% or CA 30% with TPC 30%.

**Figure 9:**
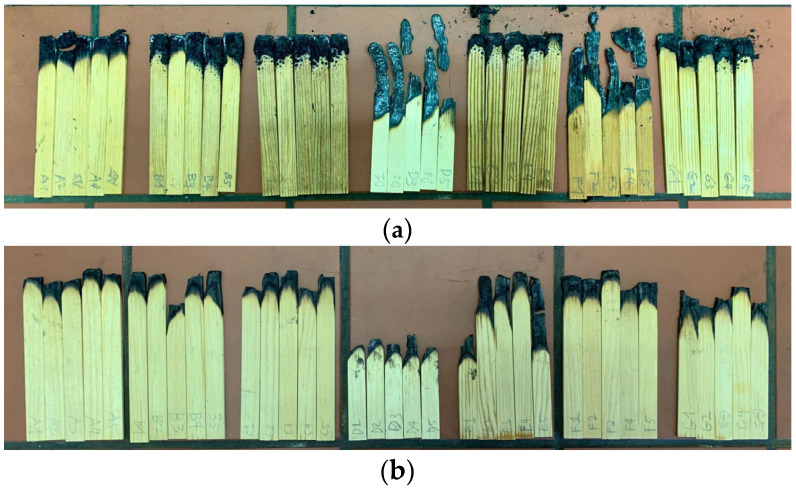
Burner specimens (**a**) before and (**b**) after EN 84:2020 procedure. Specimens left to right order: SorCA 30% combined with TPC 10%, SorCA 30% combined with TPC 20%, SorCA 30% combined with TPC 30%, control, TPC 30%, SorCA 30%, and CA 30% combined with TPC 30%.

Figure 9 visually presents these differences in combustion aspects before and after leaching. This indicates that the incorporation of SorCA may help mitigate the effects of leaching to some extent. A similar pattern was reported in a study by Kurkowiak [10].

#### 3.2.2. Single-Flame Test

The single-flame test lasted 120 s per specimen, considering their volume and weight. The standard 30 s test prescribed by ISO 11925-2:2020 [76] was insufficient to discern differences. None of the treated specimens ignited after 120 s of exposure to the flame, except for the untreated specimen, as shown in Table 3. The untreated specimen exhibited the greatest increase in the combustion area (soot cone height).

**Figure 10:**
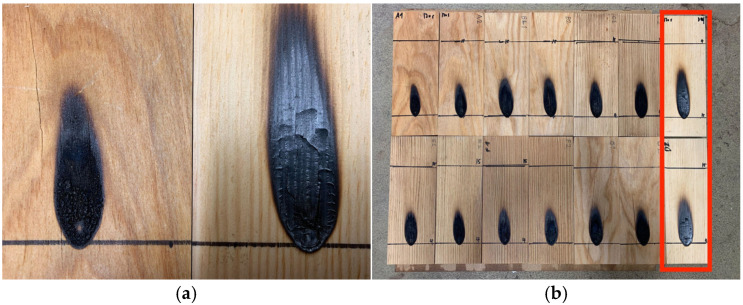
Single-flame test results and soot cone on the specimens: (**a**) treated (**left**) and untreated (**right**); (**b**) all specimens, with untreated specimens on the right end.

As depicted in Figure 10a, there is a noticeable visual distinction between the treated and untreated specimens. The treated specimens exhibit a small amount of intumescent carbonized layer on the surface, while the untreated specimens show typical shrinkage. This suggests a fire-resistant property conferred by the treatment agent, indicating penetration into the wood. However, this method may not directly confirm the adequacy of fixation. The influence of absorbed moisture in the specimens is likely minimal.

Although the test did not strictly adhere to the test standard by adjusting the test duration [102,103], the flame-retardant performance, as shown in Figure 10b, is considered robust, given that consistent results were observed at longer test times. Since no distinction was observed between treated specimens, this test might not be completely adequate to capture such differences. Thus, it is recommended to use smaller specimens or adjust the combustion time in future experiments.

#### 3.2.3. Cone Calorimeter

The cone calorimeter test is a crucial method in fire engineering, simulating radiant heat transfer in actual fire scenarios to assess a material’s combustion characteristics and flame retardancy performance [7,82,104].

Typically, the combustion pattern of wood exhibits two peaks [11,22,42,105,106]. The first peak signifies the rapid combustion of volatile materials post-ignition, where pyrolysis products undergo oxidation. Subsequently, a charcoal layer forms, acting as a protective barrier against heat, while the heat release rate (HRR) decreases due to reduced heat and oxygen transfer. The second peak represents the highest HRR, occurring as flames continue to penetrate the porous carbonized layer. This allows more air into the wood, reigniting combustion as exposed surfaces release combustible gasses. The flame progresses through the wood’s core, eventually leading to complete combustion. The HRR then diminishes gradually until the flame extinguishes [7,15,22,41,42,82,94].

**Figure 11:**
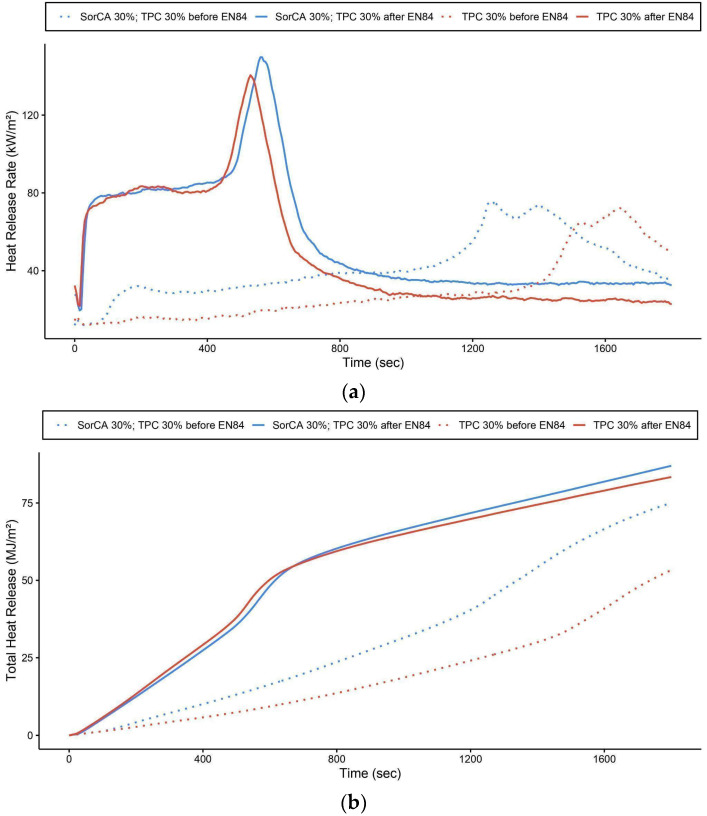
Cone calorimeter results: (**a**) heat release rate (HRR) (kW/m^2^) and (**b**) total heat release (THR) (MJ/m^2^) of specimens before and after EN 84:2020 procedure.

Comparative tests were also conducted before and after EN 84:2020 leaching (Figure 11a,b). The results of the untreated specimens aligned with findings from other flame retardancy studies, showing the rapid formation of charred residues [10] with substantial charcoal production [42]. Additionally, as described in Table 4, the time to peak HRR was delayed, indicating lower peak HRR values and reduced fire growth rates [22,37,82]. Due to reduced ignitability [7], the burning pattern of the wood was altered [42].

SorCA 30% with TPC 30%, which was not leached, had a moisture content of 10.8% (±0.2), whereas TPC 30%, also not leached, had a moisture content of 20.8% (±0.3), highlighting the hygroscopic character of TPC. Both specimens, SorCA 30% with TPC 30% and TPC 30%, exhibited rapid intumescent char formation, which impedes ignition and results in weak combustion. Consequently, the igniter had to be manually relocated during testing. Some specimens were self-extinguished and reignited, as reflected in a wide standard deviation of mass loss. During peak HRR, intumescent char was notably significant. Thus, while TPC demonstrates distinguished flame-retardant properties, its fixation post-leaching remains uncertain.

**Figure 12:**
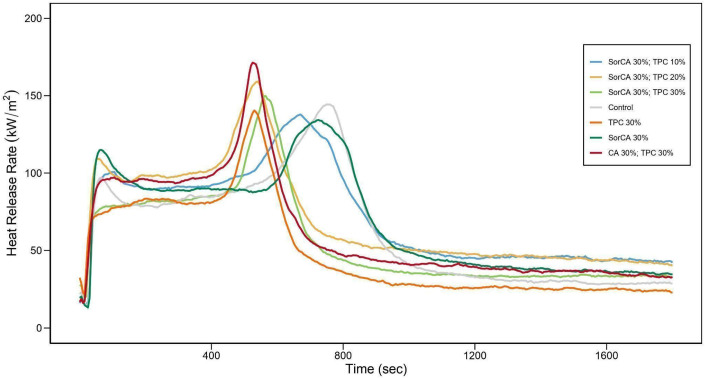
Cone calorimeter’s heat release rate (HRR) (kW/m^2^) of specimens after EN 84:2020 procedure.

Figure 12 depicts the heat release rate (HRR) graph of the specimens post-leaching. Specimens exhibiting significant leaching demonstrate shorter times to reach the maximum peak and higher maximum HRR values compared to untreated specimens. Conversely, specimens with minimal leaching show HRR values similar to those of untreated specimens. The shorter time to the maximum HRR [82], higher HRR values, and shorter ignition times [107] indicate increased fire risk [42,104]. As shown in Table 5, TPC-treated specimens quickly reach their maximum HRR after leaching and show a high total heat release, indicating a relative vulnerability to fire.

These results suggest the inadequate fixation of TPC within the wood, leading to a relatively poor flame-retardant performance compared to untreated wood. The lower moisture content in SorCA 30% with TPC 30% and SorCA 30% may have influenced the experimental outcomes [54,108], and cracks could have also impacted the results [15,82], although this was not observed in this case. The burner test demonstrated some level of flame retardancy even after leaching, possibly due to varying heat fluxes [109,110].

### 3.3. Mechanical Properties

#### 3.3.1. Three-Point Bending Test

**Figure 13:**
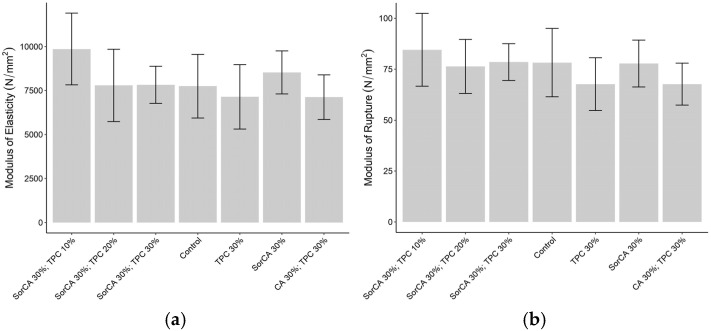
Three-point bending test results (N/mm^2^) without EN 84:2020 procedure: (**a**) modulus of elasticity (MOE); (**b**) modulus of rupture (MOR).

Figure 13a illustrates the modulus of elasticity (MOE), and Figure 13b presents the results of the modulus of rupture (MOR). Both graphs displayed values within the same range, showing no significant changes due to the treatment agents. Similar trends of increases or decreases in values by treatment group, compared to untreated wood, were also observed in other studies [8,22,23,25,35,111,112,113].

Therefore, cellulose degradation is not likely to have influenced the results for treated wood, as they are not substantially different from those of untreated wood. Additionally, hydrolysis under acidic conditions is an unlikely factor since only the SorCA single treatment is relevant, and hygroscopicity is also improbable because moisture seems to be deposited in the lumen.

Another consideration is the impact of dual forces, such as in the flexural strength experiment, which highlights the anisotropy of wood [8,101]. This indicates that variations in the compressive and tensile strengths could influence both the modulus of elasticity (MOE) and modulus of rupture (MOR). For treated wood, the compressive strength tends to increase, while the tensile strength decreases [14,101]. When the treatment increases the compressive strength but reduces the tensile strength, the resulting values might be offset, resembling those of untreated wood [114]. Conversely, if the compressive strength also decreases, both the MOE and MOR may decline, as observed in CA 30% with TPC 30%.

#### 3.3.2. Impact Bending Strength Test

The impact bending strength (IBS) test assesses the toughness and resistance of wood to sudden impacts. It measures the energy absorbed by the wood until it breaks or fractures, indicating its ability to withstand abrupt forces without failing [115,116].

**Figure 14:**
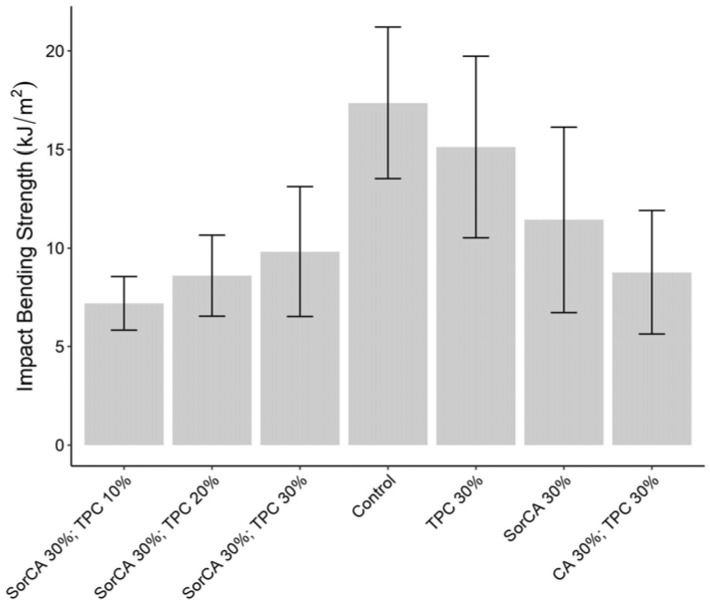
Impact bending strength results (kJ/m^2^) without EN 84:2020 procedure.

The results depicted in Figure 14 show that the mechanical properties of the treated specimens are generally lower compared to those of the untreated specimens. This phenomenon is consistent with findings from other studies on chemical modifications, where the dynamic mechanical strength tends to decrease [6,9,14,25,114,117,118].

The potential causes include the reduced flexibility of cellulose fibers due to resin-induced cross-linking [8,14,22] and the hydrolysis of wood polymer components in acidic conditions [8,25,119]. This leads to a diminished capacity for energy dispersion [111], resulting in a brittle effect on the wood [118]. Additionally, side effects from suboptimal reaction conditions, such as thermosetting temperatures [8], reduced resin content in the cell wall [9], the properties and concentration of the treatment solution [6,14], and heterogeneous cross-sectional treatment [35], should be considered. Given that their strength is significantly lower than that of untreated wood, the likelihood of degradation of the treated specimens is high.

### 3.4. Chemical Property Analysis

#### FTIR-ATR Analysis

**Figure 15:**
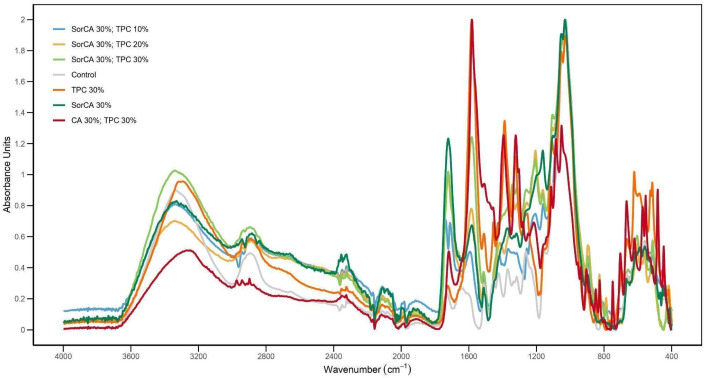
FTIR spectra of untreated wood and treated wood without EN 84:2020.

Figure 15 confirms the presence of esterification and the chemical basis of polymerization by curing the impregnated specimen in an oven at 140 °C for 24 h and analyzing the polymer in mid-infrared light.

The control shows a general band of untreated wood [120,121]. Peaks around 1730 cm^−1^ in untreated wood correspond to C=O basic vibrations of the esters and acetyl groups of xylan [122]. Peaks around 1250 cm^−1^ are due to the carboxyl functional groups of xylan and hemicellulose [123,124,125]. As it is treated, a peak of around 1700 cm^−1^ is created, indicating that esterification proceeds according to the C=O stretch of CA. Similarly, symmetrical stretching and vibration of the ester around 1200–1000 cm^−1^ can be confirmed [88,126]. The region of 3500 to 3100 cm^−1^ may be attributed to the asymmetric stretching vibrations of the O-H group occurring in the wood’s main chemical component, possibly due to the wood’s involvement in the grafting reaction [127,128,129].

## 4. Conclusions

This paper focused on the fixation of tripotassium Citrate (TPC) as a flame-retardant agent within the wood matrix using SorCA thermosetting resin. To evaluate the stabilization of the fire-retardant agent, analyses were conducted on the chemical properties, dimensional stability, mechanical properties, flame retardancy, and leaching tests. Chemical analysis confirmed the possibility of esterification, and the combination of TPC and SorCA showed promising solution uptake post-impregnation. The cell wall bulking value was higher than those of other solutions, resulting in positive weight percent gain (WPG) outcomes, while the anti-swelling efficiency (ASE) demonstrated the effectiveness of the combination. As observed in similar impregnation studies, the dynamic mechanical strength tended to decrease; however, static strength was in the same range as untreated wood and, thus, remained comparable. This suggests that while the modified wood may not perform optimally under dynamic conditions, it could still be suitable for applications where static loads are predominant. Further studies should aim to refine treatment techniques to improve the dynamic mechanical properties while maintaining the static strength. Flame retardancy tests revealed excellent results for specimens treated with TPC, with the standalone TPC treatment yielding the most effective outcomes. However, the leaching tests highlighted a challenge, reaffirming that the leaching of flame retardants remains an obstacle to be addressed. Notably, the burner test demonstrated improved flame retardancy for the combination of TPC and SorCA, even under the EN 84:2020 leaching regime, although this positive outcome was not observed in cone calorimetry. Dimensional stability, mechanical properties, and flame retardancy are essential properties for wood as a material, and they must be addressed in an eco-friendly and sustainable manner. This study reconfirms the need for various approaches to balance all three properties effectively.

The bio-based TPC exhibited a noteworthy flame-retardant performance. Economically, TPC and SorCA offer a cost-effective, widely available alternative to conventional synthetic flame retardants. Their use meets the demand for environmentally friendly materials while enhancing wood durability and safety. To address the challenges identified in this study, particularly in the fixation and performance of flame retardants, future research should focus on exploring other bio-based compounds that might offer stronger bonding capabilities with wood. Additionally, optimizing the curing conditions and investigating alternative curing resins could potentially enhance the chemical interactions and stability of the flame retardant within the wood matrix. Furthermore, developing new modification methods, such as thermal treatments or advanced polymerization techniques, could lead to more robust flame-retardant systems. Such research could pave the way for more efficient, durable, and sustainable wood-modification agents that align with the growing demand for eco-friendly building materials.

## Figures and Tables

**Table 1:** Solution formulation for each collective and its pH value.

Chemicals	Additional	pH Value
SorCA (1:3; 30%)	TPC (10%)	2.97
SorCA (1:3; 30%)	TPC (20%)	4.12
SorCA (1:3; 30%)	TPC (30%)	4.81
Control	n/a	n/a
TPC (30%)	n/a	8.62
SorCA (1:3; 30%)	n/a	1.49
Citric acid (30%); TPC (30%)	n/a	4.90

**Table 2:** Wood specimen preparation for tests.

Specimen Size (mm^3^)	Test type	Replicates per Collective
20 × 20 × 10	Dimensional stability	10
4 × 13 × 125	Flame retardant	5
20 × 90 × 250	Flame retardant	2
18 × 100 × 100	Flame retardant	5
10 × 10 × 160	Mechanical properties	20

**Table 3:** Single-flame test results.

Designation	Ignition (Yes/No)	Flame-Out Time (s)	Soot Cone Height (mm)	Pass/Fail * (Yes/No)
SorCA 30%; TPC 10%	No	n/a	63	Yes
SorCA 30%; TPC 20%	No	n/a	78	Yes
SorCA 30%; TPC 30%	No	n/a	72.5	Yes
Control	Yes	18	101.5	Yes
TPC 30%	No	n/a	65.6	Yes
SorCA 30%	No	n/a	72	Yes
CA 30%; TPC 30%	No	n/a	74.5	Yes

* According to ISO 11925-2:2020, the test is considered acceptable if the flame extinguishes within 30 s after removing the burner, without exceeding a height of 150 mm.

**Table 4:** Cone calorimeter test results before and after EN 84:2020 procedure.

Designation	Ignition Time (s)	1PHRR	Time to 1PHRR (s)	Peak HRR	Time to Peak HRR (s)	THR at 600 s	Mass Loss (%)
SorCA 30%; TPC 30% before EN 84	88 (±18)	32.1 (±4.6)	190	75.9 (±1.3)	1260	16.4 (±1.5)	67.1 (±2)
SorCA 30%; TPC 30% after EN 84	22 (±4)	80.2 (±5.1)	145	149.8 (±28.5)	565	48.2 (±8.8)	78.3 (±3.7)
TPC 30% before EN 84	161.3 (±7)	16.4 (±18)	180	72 (±21.4)	1640	9.3 (±0.9)	44.6 (±31.2)
TPC 30% after EN 84	15.6 (±3)	78.1 (±2.8)	120	140.5 (±18.9)	530	50.2 (±4.5)	76.6 (±4.3)

**Table 5:** Cone calorimeter test results after EN 84:2020 procedure.

Designation	Ignition Time (s)	1PHRR	Time to 1PHRR (s)	Peak HRR	Time to Peak HRR (s)	THR at 600 s	Mass Loss (%)
SorCA 30%; TPC 10%	28.8 (±6)	100.7 (±1.5)	105	137.9 (±8.7)	670	54.6 (±2.9)	86.2 (±2.5)
SorCA 30%; TPC 20%	18.2 (±5)	109.1 (±2.7)	60	159.1 (±16)	540	63.8 (±1.9)	86.9 (±1.6)
SorCA 30%; TPC 30%	22 (±4)	80.2 (±5.1)	145	149.8 (±28.5)	565	58.2 (±8.8)	88.3 (±3.7)
Control	32.8 (±4)	97.1 (±3.9)	65	144.4 (±7.1)	750	49.1 (±3.6)	91.4 (±9.3)
TPC 30%	15.6 (±3)	78.1 (±2.8)	120	140.5 (±18.9)	530	60.2 (±4.5)	86.6 (±4.3)
SorCA 30%	28.4 (±2)	115 (±3.9)	65	134.3 (±3.4)	725	52.9 (±1.0)	80.6 (±6.8)
CA 30%; TPC 30%	20.8 (±12)	97.2 (±2.8)	105	171.4 (±16.1)	525	61.3 (±2.8)	89.7 (±4.6)

## Data Availability

The original contributions presented in the study are included in the article, further inquiries can be directed to the corresponding author.
